# Author Correction: Ampere-hour-scale soft-package potassium-ion hybrid capacitors enabling 6-minute fast-charging

**DOI:** 10.1038/s41467-023-43045-0

**Published:** 2024-01-08

**Authors:** Huanxin Li, Yi Gong, Haihui Zhou, Jing Li, Kai Yang, Boyang Mao, Jincan Zhang, Yan Shi, Jinhai Deng, Mingxuan Mao, Zhongyuan Huang, Shuqiang Jiao, Yafei Kuang, Yunlong Zhao, Shenglian Luo

**Affiliations:** 1 State Key Laboratory for Chemo/Biosensing and Chemometrics, College of Chemistry and Chemical Engineering, Hunan University, 410082 Changsha, Hunan China; 2 Department of Engineering, University of Cambridge, 9 JJ Thomson Avenue, Cambridge, CB3 0FA UK; 3 Department of Chemistry, Physical & Theoretical Chemistry Laboratory, University of Oxford, South Parks Road, Oxford, OX1 3QZ UK; 4 Electrochemical Innovation Lab, Department of Chemical Engineering, University College London, London, WC1E 7JE UK; 5 Dyson School of Design Engineering, Imperial College London, London, SW7 2BX UK; 6 Advanced Technology Institute, University of Surrey, Guildford, Surrey GU2 7XH UK; 7 Department of Chemical & Process Engineering, University of Surrey, Guildford, GU2 7XH UK; 8 College of Materials and Metallurgy, Guizhou University, 550025 Guiyang, China; 9 King’s College London, London, SE1 1UL UK; 10 Department of Electrical and Electronic Engineering, Imperial College London, London, SW7 2AZ UK; 11 State Key Laboratory of Advanced Metallurgy, University of Science and Technology Beijing, 100083 Beijing, China

**Keywords:** Energy storage, Materials for energy and catalysis

Correction to: *Nature Communications* 10.1038/s41467-023-42108-6, published online 12 October 2023

In this article several affiliations were incorrect.

The affiliation details for Huanxin Li were incorrectly given as ‘Advanced Technology Institute, University of Surrey, Guildford, Surrey GU2 7XH, UK’ and ‘State Key Laboratory of Advanced Metallurgy, University of Science and Technology Beijing, Beijing 100083, China’ but should have been ‘Department of Engineering, University of Cambridge, 9 JJ Thomson Avenue, Cambridge CB3 0FA, UK’ and ‘Department of Chemistry, Physical & Theoretical Chemistry Laboratory, University of Oxford, South Parks Road, Oxford OX1 3QZ, UK’.

The affiliation details for Yi Gong were missing the affiliation of ‘Advanced Technology Institute, University of Surrey, Guildford, Surrey GU2 7XH, UK’.

The affiliation details for Kai Yang were incorrectly given as ‘Dyson School of Design Engineering, Imperial College London, London SW7 2BX, UK’, but should have been “Advanced Technology Institute, University of Surrey, Guildford, Surrey GU2 7XH, UK’.

The affiliation details for Boyang Mao and Jincan Zhang were incorrectly given as ‘Advanced Technology Institute, University of Surrey, Guildford, Surrey GU2 7XH, UK’, but should have been ‘Department of Engineering, University of Cambridge, 9 JJ Thomson Avenue, Cambridge CB3 0FA, UK’.

The affiliation details for Yan Shi were incorrectly given as ‘Department of Engineering, University of Cambridge, 9 JJ Thomson Avenue, Cambridge CB3 0FA, UK’, but should have been ‘College of Materials and Metallurgy, Guizhou University, 550025 Guiyang, China’.

The affiliation details for Jinhai Deng were incorrectly given as ‘College of Materials and Metallurgy, Guizhou University, Guiyang 550025, China’, but should have been ‘King’s College London, London SE1 1UL, UK’.

The affiliation details for Mingxuan Mao were incorrectly given as ‘King’s College London, London SE1 1UL, UK’, but should have been ‘Department of Electrical and Electronic Engineering, Imperial College London, London SW7 2AZ, UK’.

The affiliation details for Shuqiang Jiao were incorrectly given as ‘Department of Electrical and Electronic Engineering, Imperial College London, London SW7 2AZ, UK’, but should have been ‘State Key Laboratory of Advanced Metallurgy, University of Science and Technology Beijing, 100083 Beijing, China’.

Incorrect additional affiliation details for Yunlong Zhao were given as ‘Department of Chemistry, Physical & Theoretical Chemistry Laboratory, University of Oxford, South Parks Road, Oxford OX1 3QZ, UK’.

The original article has been corrected. The HTML has been updated to include a corrected version of the Supplementary Information.

### Supplementary information

Updated Supplementary Information

