# Supplementary material for: Ampere-hour-scale soft-package potassium-ion hybrid capacitors enabling 6-minute fast-charging
Source: Nat Commun. 2023 Oct 12;14:6407. doi: 10.1038/s41467-023-42108-6 (PMC10570334; doi:10.1038/s41467-023-42108-6)
Supplement: Supplementary file 1 — Supporting Information [file 41467_2023_42108_MOESM1_ESM.pdf]

Supplementary Information

Ampere-hour-scale soft-package potassium-ion hybrid capacitors enabling 6-minute fast-charging

Huanxin Li,[1, 2, 3, 4#] Yi Gong,[5, 6#], Haihui Zhou,[1*] Jing Li,[7] Kai Yang,[6] Boyang Mao,[2] Jincan Zhang,[2] Yan Shi,[8] Jinhai Deng,[9] Mingxuan Mao,[10] Zhongyuan Huang,[1] Shuqiang Jiao,[11*] Yafei Kuang,[1] Yunlong Zhao,[5*] Shenglian Luo[1*]

Supplementary Note 1: Calculating capacitive vs diffusive current.

To verify the contribution of pseudocapacitive behaviour, further analysis was conducted as follows. The relationship between the peak current density (i) and the sweep rate (v) could be described as the following equations[1]:

$$\mathrm{i} = \mathrm{a}v^{\mathrm{b}} \quad (1)$$

$$\log(i) = \mathrm{b} * \log(v) + \log(\mathrm{a}) \quad (2)$$

where the b value could be determined by the slope of linear fitted log(i)-log (v) curves.

Typically, when the b value is approaching 0.5, it indicates that the intercalation of potassium ion is mainly determined by the diffusion process, while the b value gets close to 1.0, the potassium storage is dominated by the pseudocapacitor behaviours, such as surface-controlled capacitive process or intercalation without phase transition.

The quantitative calculation of capacitive contribution ratio during the potassium-ion storage process was conducted by the following equation[2]:

$$i(\mathrm{V}) = \mathrm{k}_1 v^{1/2} + \mathrm{k}_2 v \quad (3)$$

$$i/v^{1/2} = \mathrm{k}_1 + \mathrm{k}_2 v^{1/2} \quad (4)$$

where i (V) is the current density at a certain potential, v is the scan rate, k_1 and k_2 are constants at a certain potential. The values of k_1 and k_2 can be calculated through Eq. (3) and (4), which correspond to the slope and intercept of the fitted lines of $v^{1/2}$ versus i /$v^{1/2}$ plots.

Supplementary Figures

CV tests for the capacitive behaviours

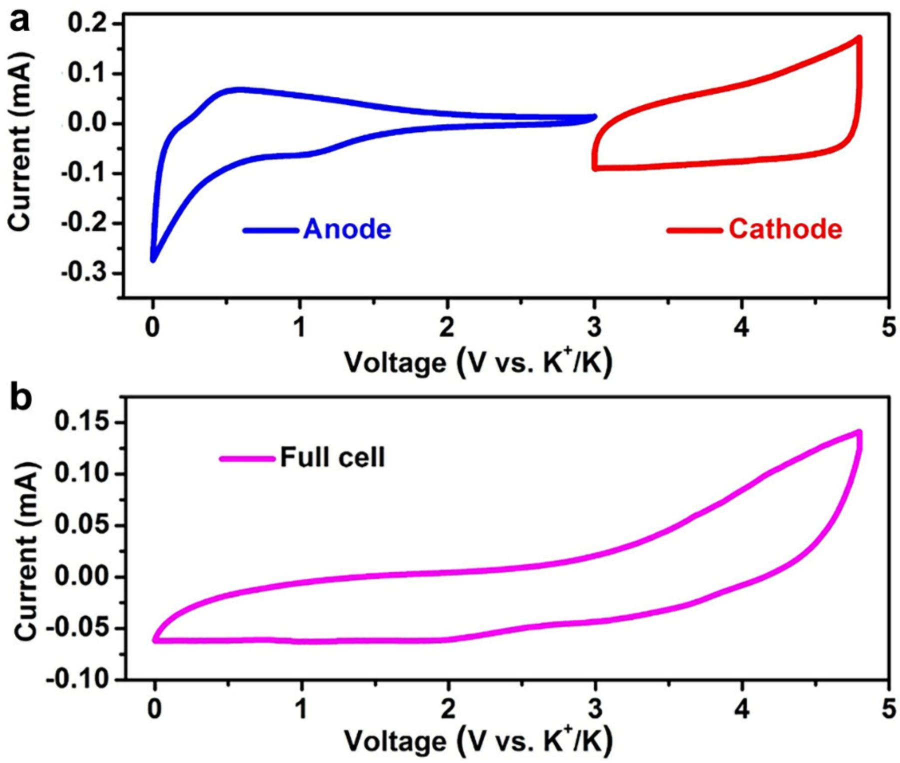

Supplementary Fig. 1. a Schematic of the mechanism for potassium storage in PIBHCD. **b** The relative shapes of CV curves for full cell.

Morphologies of electrodes (Supplementary Fig. 2-7)

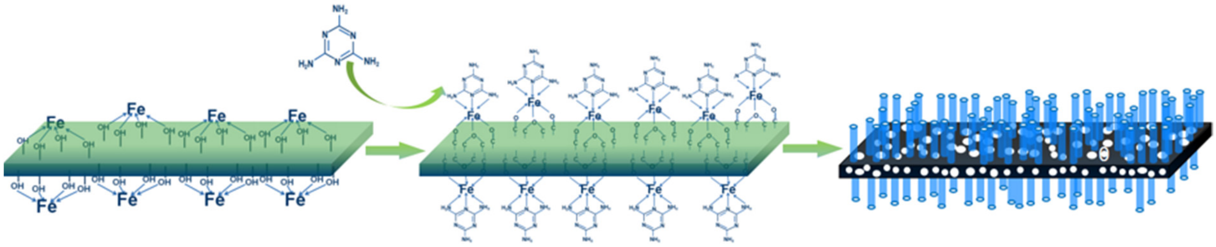

Supplementary Fig. 2 The schematic illustration of the process of preparing the N-CNTs@MC.

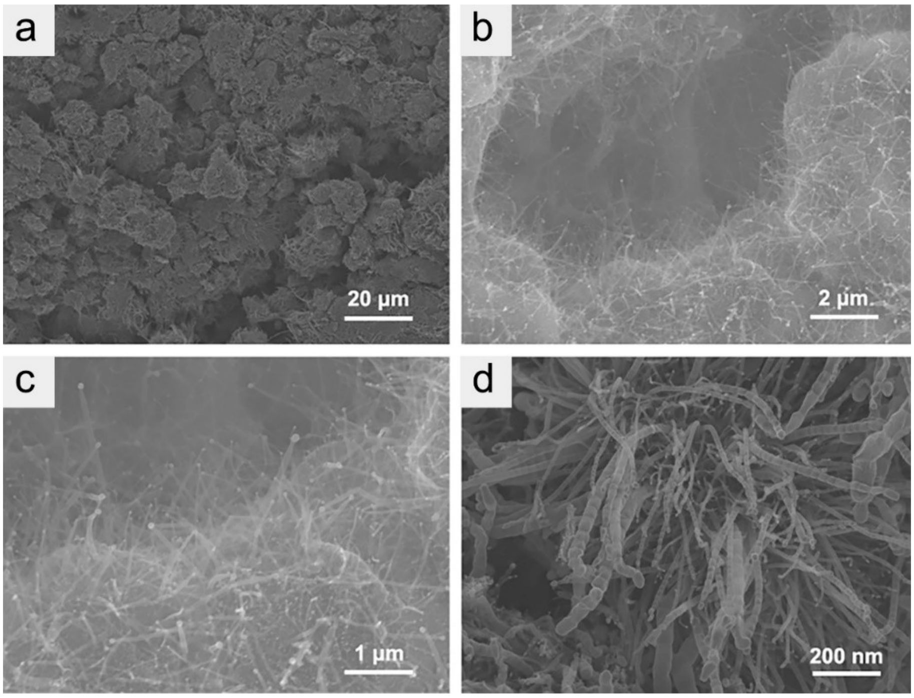

Supplementary Fig. 3. SEM images of Fe_2O_3-N-CNTs@MC at different magnification, scale bar: **a** 20 µm. **b** 2 µm. **c** 1 µm. **d** 200 nm.

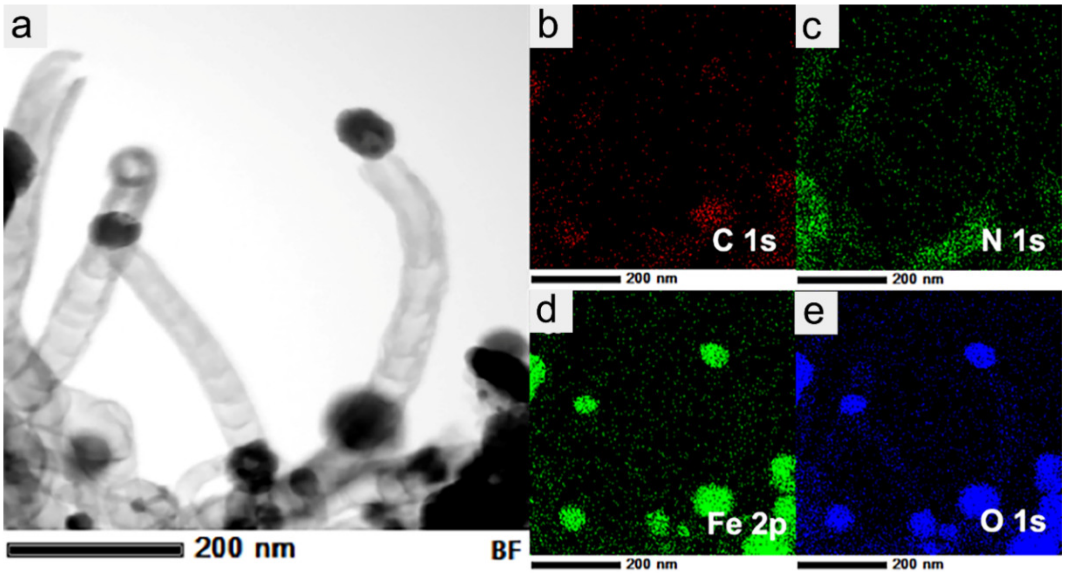

Supplementary Fig. 4. a Typical TEM images of Fe_2O_3-N-CNTs@MC and corresponding mapping images for **b** C, **c** N, **d** Fe and **e** O elements.

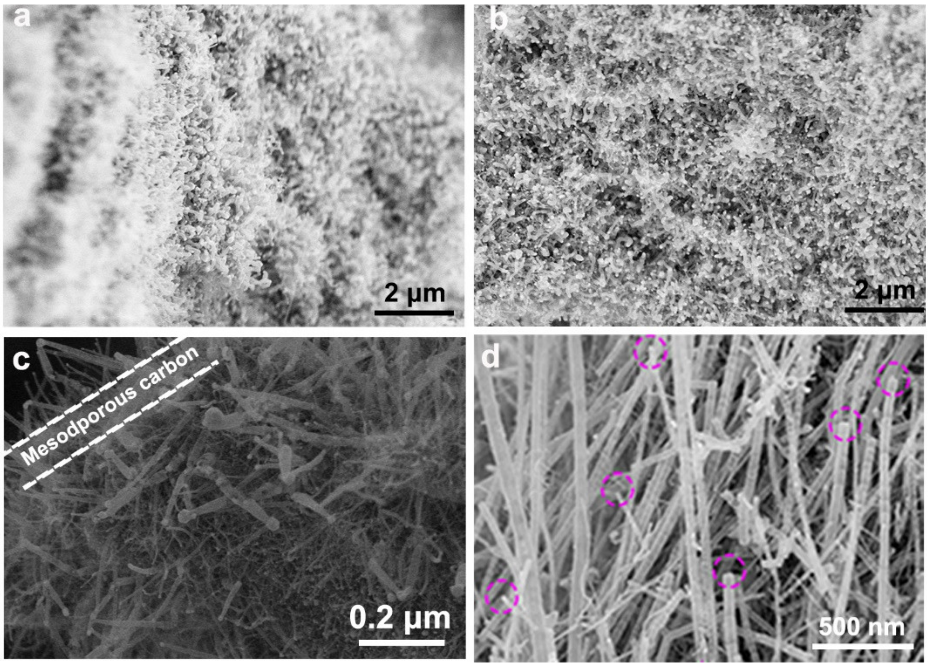

Supplementary Fig. 5. SEM images of N–CNTs@MC after acid wash and high-temperature heat treatment at different magnification, scale bar: **a** 2 µm, **b** 2 µm, **c**. 0.2 µm, **d** 500 nm (pink circle: open end of the CNTs).

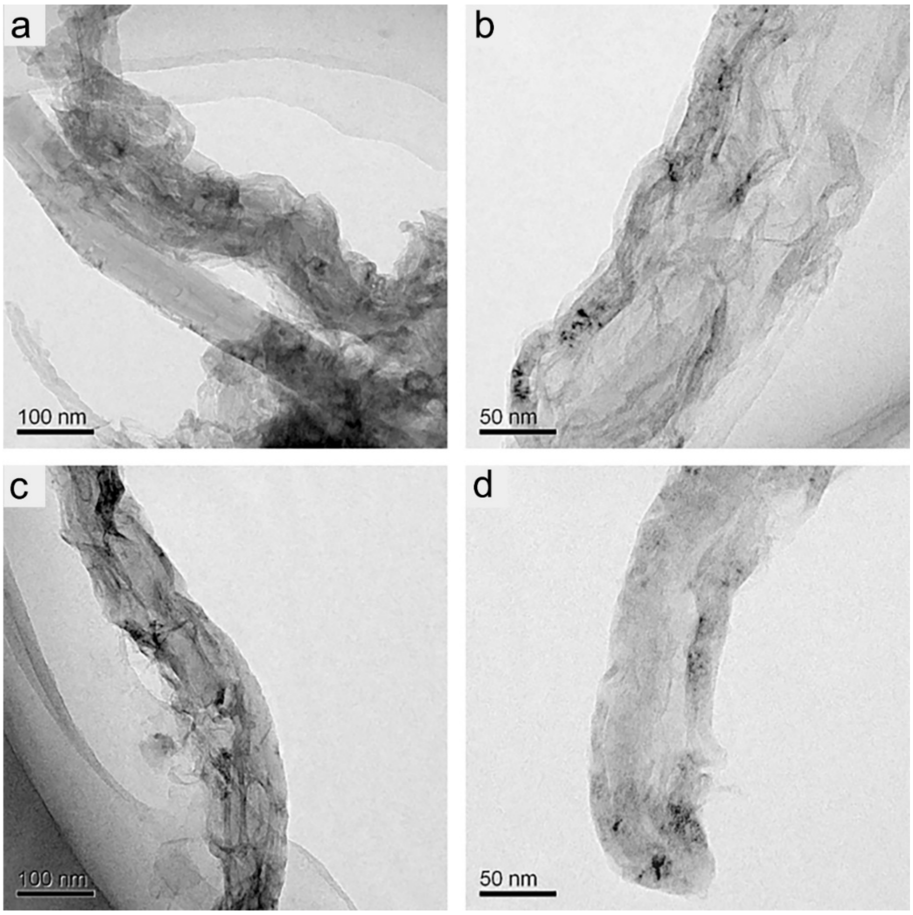

Supplementary Fig. 6. TEM images of the carbon nanotubes in N–CNTs@MC after acid washing: **a** and **b** depict N-CNTs at various locations on the MC, while **c** and **d** show individual N-CNTs in distinct regions.

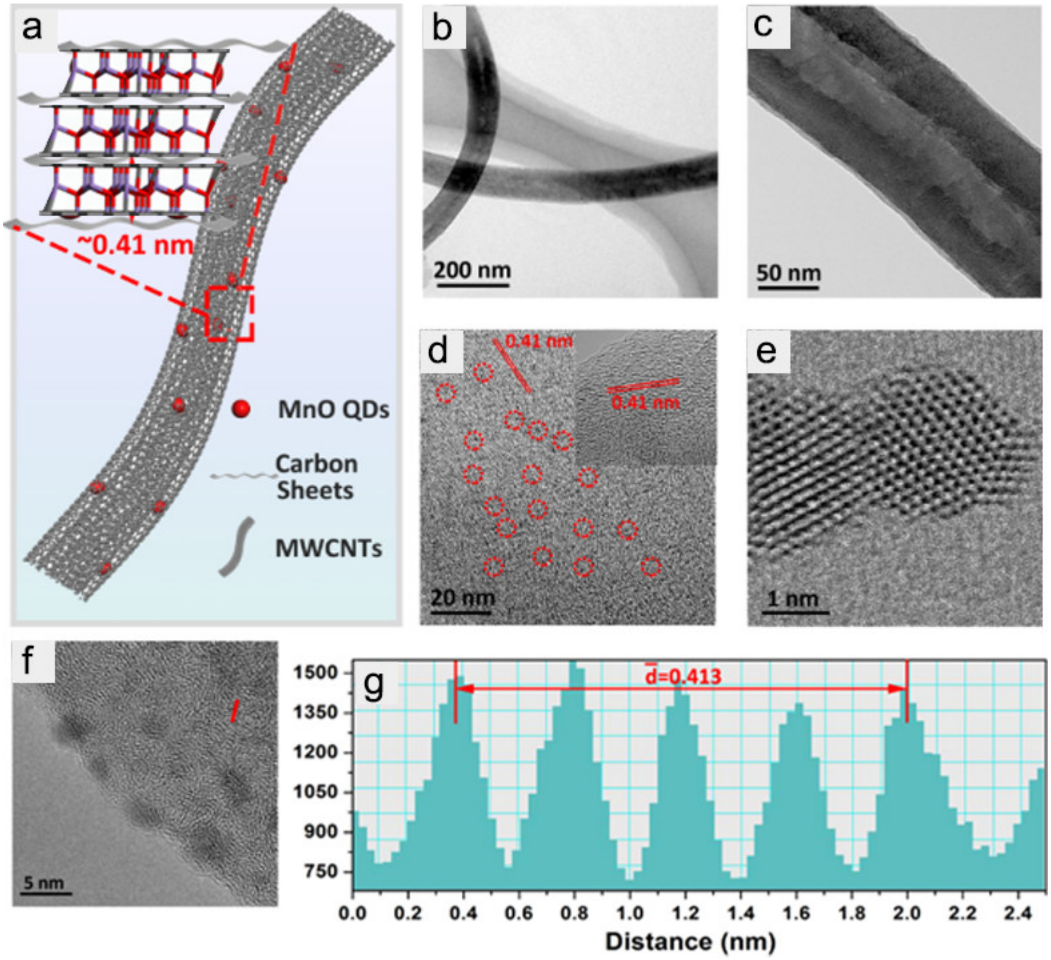

Supplementary Fig. 7. **a** Schematic diagram, **b** TEM, and **c** enlarged TEM images of MnO@CNTs; **d** High resolution TEM image on the wall of MnO@CNTs; **e** Atomic image of the MnO quantum dots; **f** TEM image of MnO@CNTs with clear diffraction fringes of carbon layer and **g** corresponding carbon layer spacing.

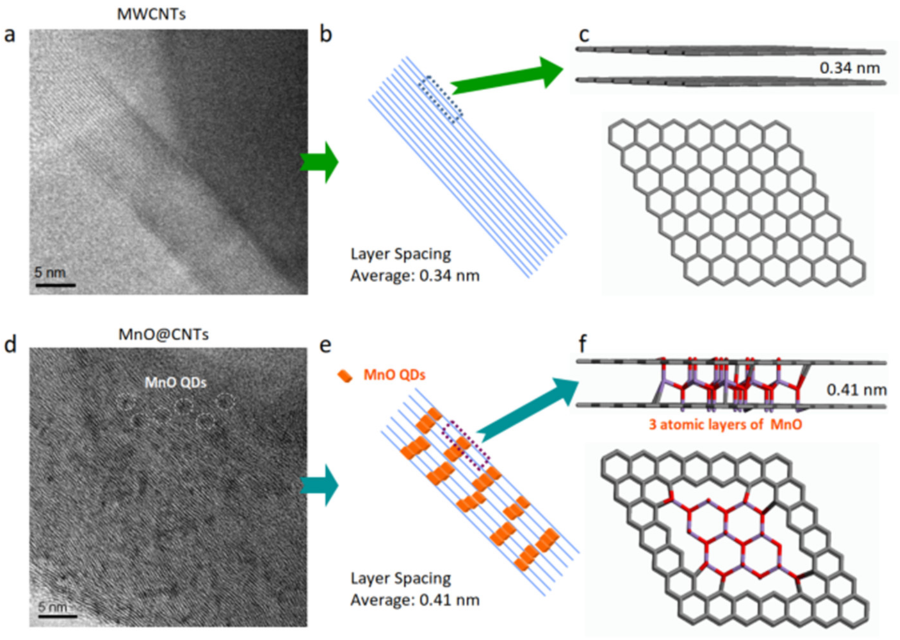

Supplementary Fig. 8. The illustration of the comparison between MWCNTs and Mno@CNTs. **a** The TEM image of the MWCNTs, **b** The schematic illustration of the layer spacing of the MWCNTs, **c** The atomic structure of the MWCNTs, **d** The TEM image of the MnO@CNTs, **e** The schematic illustration of the structure of the MnO@CNTs, **f** The atomic structure of the MnO@CNTs.

Compositions and structures (Supplementary Fig. 9-16)

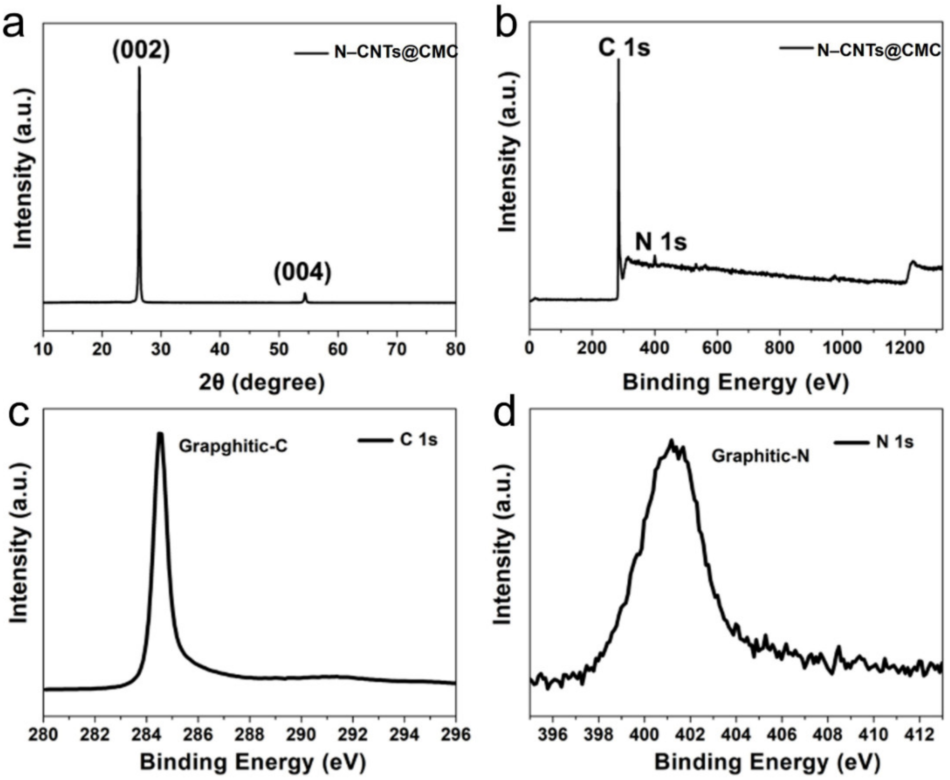

Supplementary Fig. 9. **a** XRD pattern of N–CNTs@CMC; **b** XPS survey of N–CNTs@CMC; **c** XPS high resolution peaks of C 1*s* and **d** N 1s in N–CNTs@CMC.

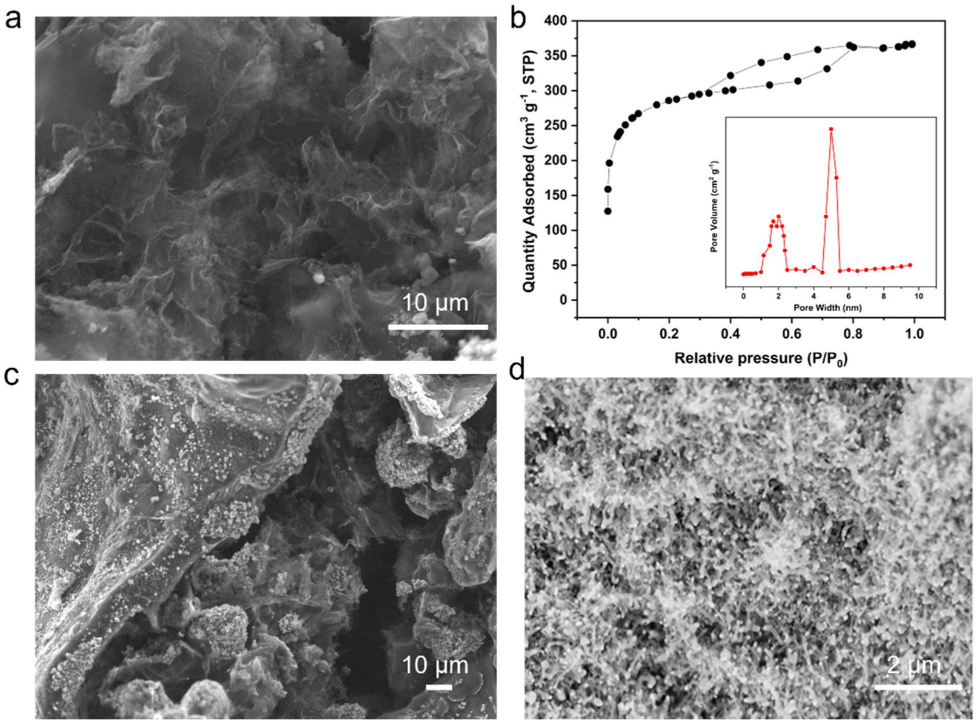

Supplementary Fig. 10. a The SEM of the MC; **b** the Nitrogen adsorption-desorption isotherms and pores size distribution of the MC; **c** The SEM of Fe_2O_3@MC; **d** the SEM of the N-CNTs@MC

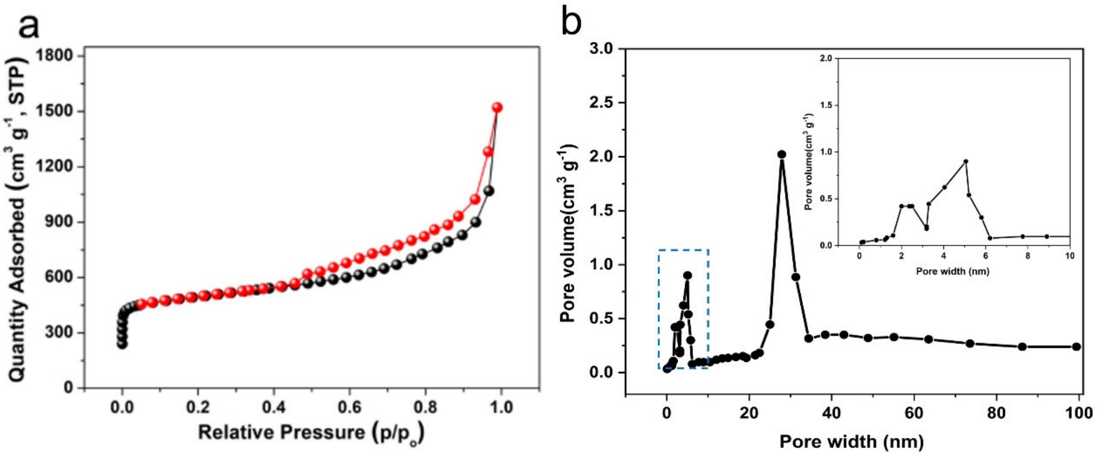

Supplementary Fig. 11. a Nitrogen adsorption-desorption isotherms and **b** pore size distributions of N–CNTs@MC.

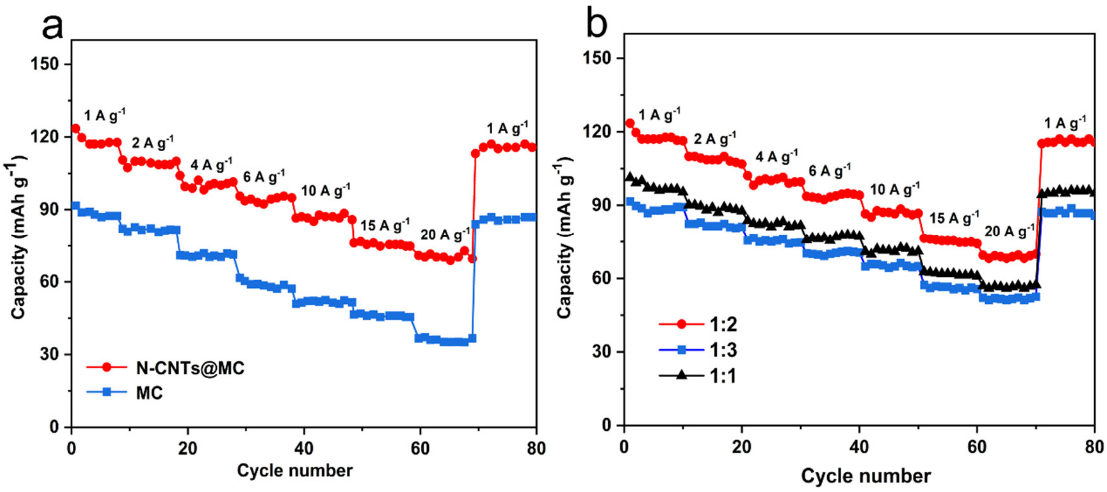

Supplementary Fig. 12. a Rate performance of the MC and N-CNTs@MC samples, **b** The comparison of the rate performance of N-CNTs@MC prepared with different ratios of MC precursor and melamine.

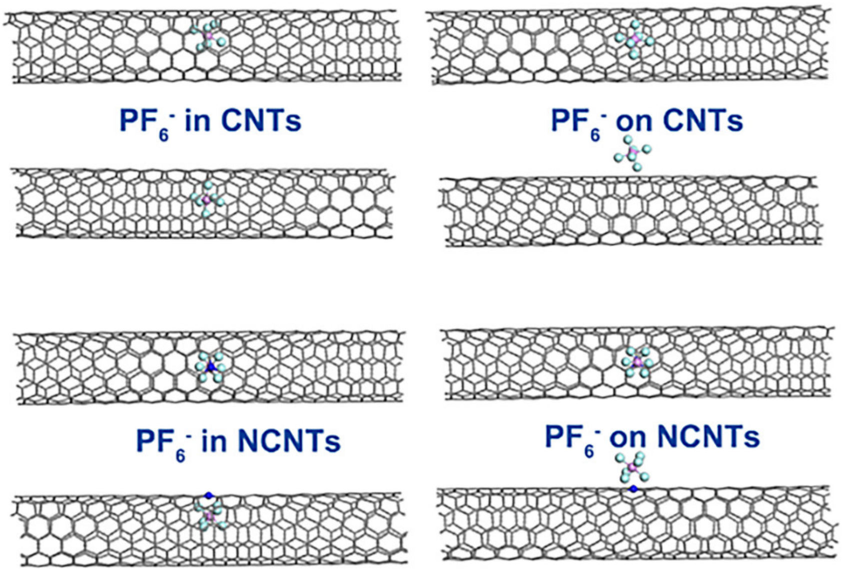

Supplementary Fig. 13. The optimized structures of adsorbed PF_6^- ions on CNT and N-CNT.

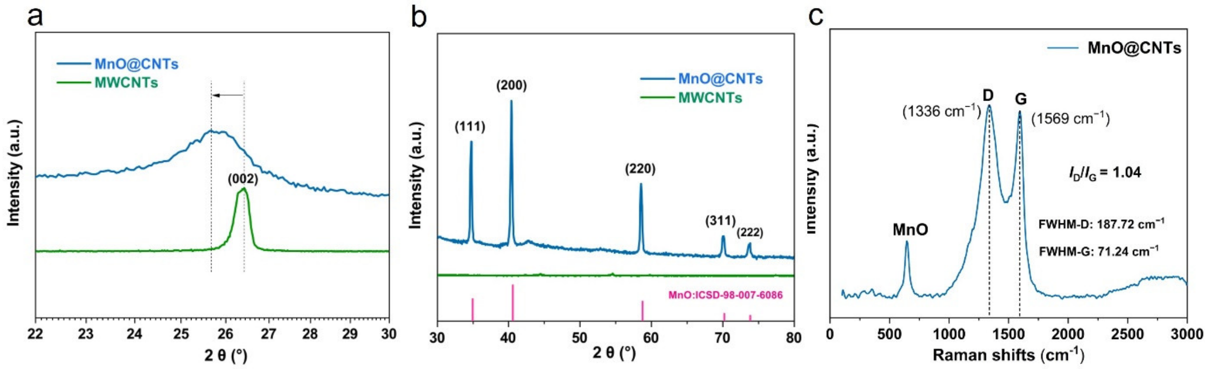

Supplementary Fig. 14. XRD patterns of MnO@CNTs and CNTs: **a** from 22° to 30°, **b** from 30° to 80°. **c** Raman spectra of MnO@CNTs.

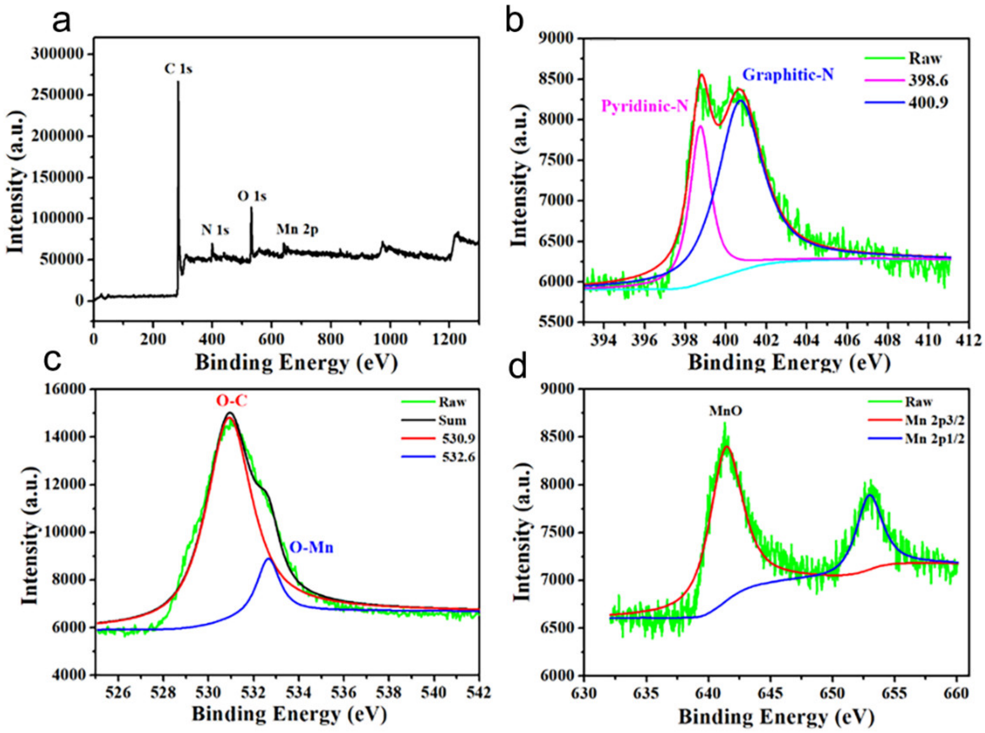

Supplementary Fig. 15. XPS survey of MnO@CNTs: **a** XPS full spectra, and high resolution XPS spectra with fittings of **b** N 1*s*, **c**. O 1*s*, **d** Mn 2*p*.

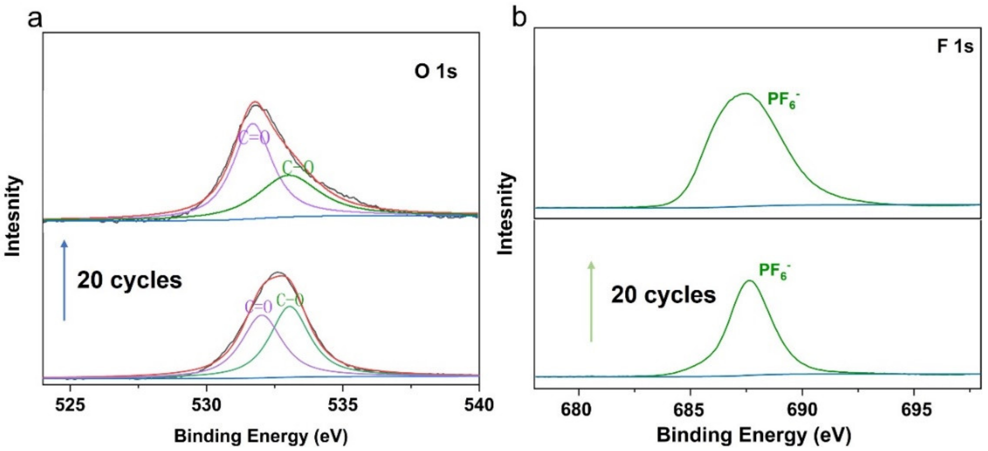

Supplementary Fig. 16. The XPS analysis of the cathode electrode before and after 20cycles: **a**. O 1*s* curves, **b** F 1*s* curves of cathode before and after 20 cycles.

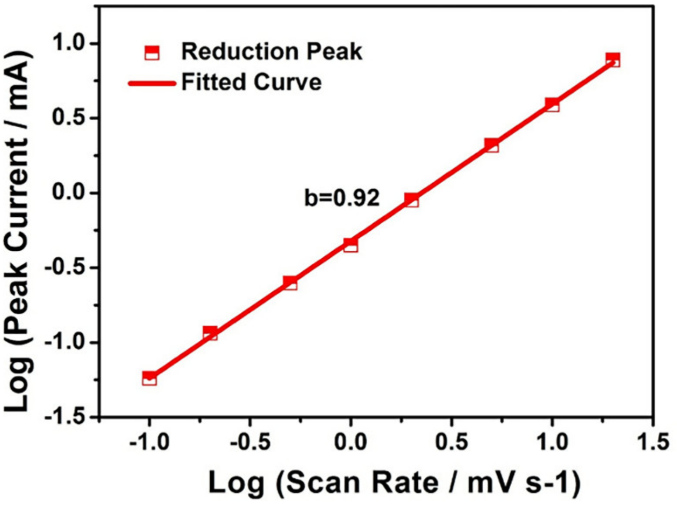

Supplementary Fig. 17. Log(i)–log(v) curves for b-value determination

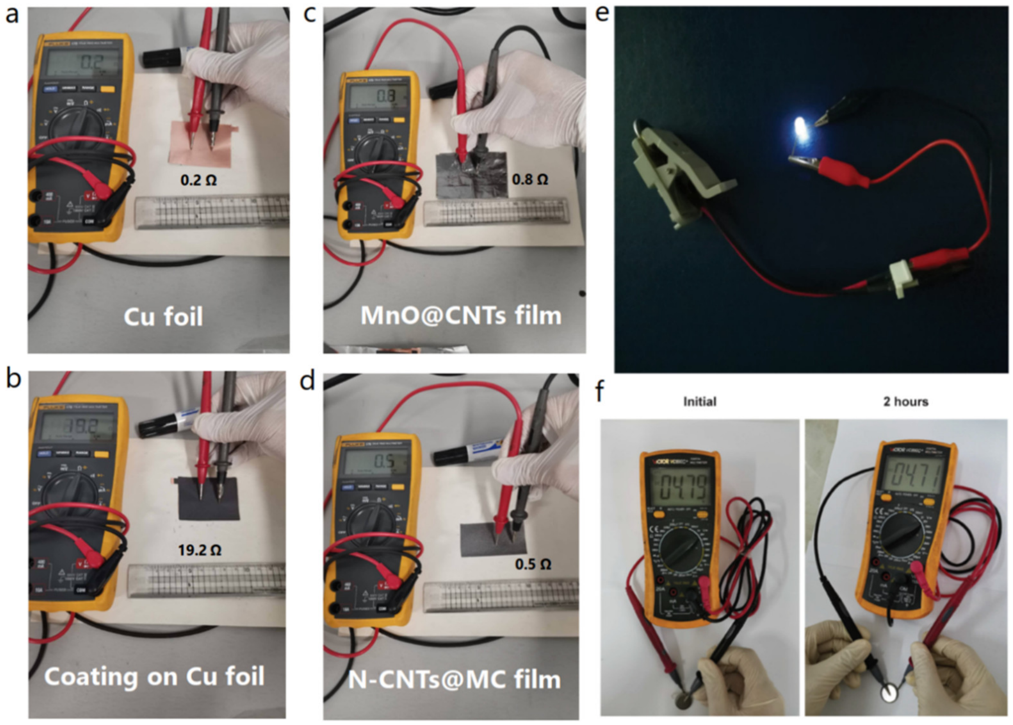

Supplementary Fig. 18. Comparison of the conductivity of **a** Cu foil, **b** the anode material coated on Cu foil, **c** N-CNTs@MC film and **d** MnO@CNTs film. **e** the photograph of the coin cell lighting a LED light, **f** the self-discharge test of the coin cell

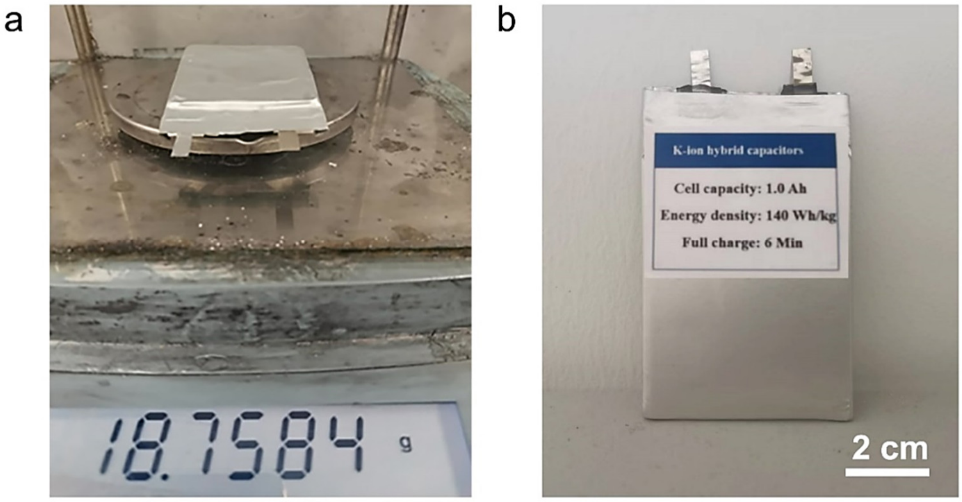

Supplementary Fig. 19. a The weight of ~1Ah PIHC. **b** The picture of the prepared 1Ah PIHC

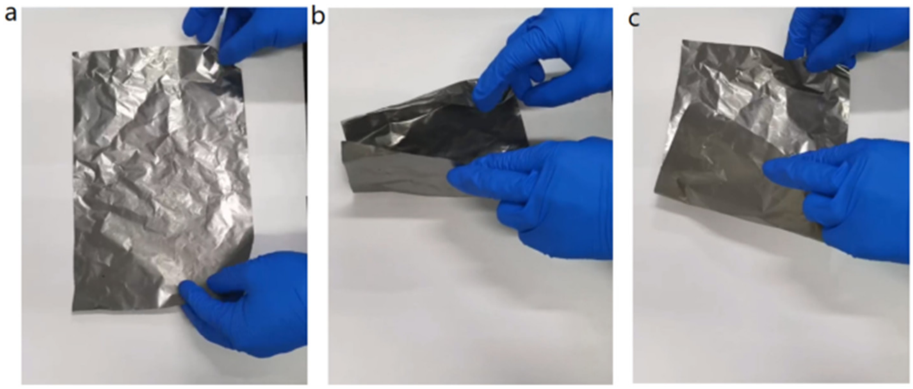

d

Hall measurements						
Sample No.	T(K)	Resistivity (ohm-cm)	Hall mobility (cm^2/Vs)	Carrier concentration ($1/cm^3$)	Hall coefficient (cm^3/C)	Conductivity (S/m)
1	300	3.13E-04	9.12E+02	2.19E+19	-2.85E-01	3.19E05
2	300	3.15E-04	9.09E+02	2.18E+19	-2.86E-01	3.17E05
3	300	3.16E-04	8.94E+02	2.22E+19	-2.82E-01	3.16E05

Supplementary Fig. 20. The photographs of **a** the N-CNTs@MC film, **b** the folded N-CNTs@MC film, **c** the N-CNTs@MC film after continuous folding. **d** The data of the Hall measurement for the N-CNTs@MC film

Supplementary Tables

Supplementary Table 1 Optimized parameters of the soft-packaged PIHC with the capacity of ~1Ah

**Parameters of winded soft-package PHICs**						
	**Cathode**	**Anode**	**Separator**	**S (cm^2)**	**Total V (cm^3)**	**Volume Density (Wh/L)**
**Area mass ($mg\ cm^{-2}$)**	30	10	1.7	**Single**	7.5	283.2
				24.8		
**Thickness (µm)**	150	40	12.0	**Stack**	**Electrolyte (mg)**	**Shell and Tags (mg)**
				10	4474.5	3186.4
**Density ($g\ cm^{-3}$)**	~20	~2.5	~1.42	**Core mass**	**Total mass (mg)**	**Energy density (Wh/Kg)**
**Mass (mg)**	7425.0	2722.5	950.0	11097.5	18758.4	140.0

Supplementary Table 2 Free energies from DFT calculations for PF_6^-, CNTs, NCNTs, PF_6^- @CNTs, PF_6^-@NCNTs, K^+, MnO@G, and K^+on G and K^+on MnO@G

**Calculation of adsorption energies (eV)**			
**PF_6^-on SCNT**			
$\boldsymbol{E}_{\mathbf{PF6^-}}$ **(eV)**	$\boldsymbol{E}_{\mathbf{CNTs}}$ **(eV)**	$\boldsymbol{E}_{\mathbf{PF6^-@CNTs}}$ **(eV)**	$\boldsymbol{\Delta E}_{\mathbf{ads\ (CNTs\ -\ PF6^-)}}$ **(eV)**
-30.92	-3103.04	-3136.15	-2.19
**PF_6^-on N-CNT**			
$\boldsymbol{E}_{\mathbf{PF6^-}}$ **(eV)**	$\boldsymbol{E}_{\mathbf{N\text{-}CNTs}}$ **(eV)**	$\boldsymbol{E}_{\mathbf{PF6^-@N\text{-}CNTs}}$ **(eV)**	$\boldsymbol{\Delta E}_{\mathbf{ads\ (N\text{-}CNTs\ -\ PF6^-)}}$ **(eV)**
-30.92	-3107.30	3148.42	-10.21
**K^+ on G**			
$\boldsymbol{E}_{\mathbf{K+}}$ **(eV)**	$\boldsymbol{E}_{\mathbf{G}}$ **(eV)**	$\boldsymbol{E}_{\mathbf{K^+@G}}$ **(eV)**	$\boldsymbol{\Delta E}_{\mathbf{ads\ (G\ -\ K+)}}$ **(eV)**
-2.36	-526.32	-529.94	-1.26
**K^+ on MnO@G**			
$\boldsymbol{E}_{\mathbf{K+}}$ **(eV)**	$\boldsymbol{E}_{\mathbf{G}}$ **(eV)**	$\boldsymbol{E}_{\mathbf{K^+@MnO@G}}$ **(eV)**	$\boldsymbol{\Delta E}_{\mathbf{ads\ (MnO@G\ -\ K+)}}$ **(eV)**
-2.36	-690.95	-698.08	-4.76

Supplementary Table 3. Comparison with the recently-reported representative PIHC

**Cathode// Anode[Ref.]**	**Voltage (V)**	**Energy density (Wh kg^{-1})**	**Power density (W kg^{-1})**	**Type of devices**
**N-CNTs@MC//MnO@CNTs** **[Our work]**	0.8-4.8	**140** Wh kg^{-1} (based on the whole weight of PIBHCD);	1400 W kg^{-1} (10 C, fully charged within **6** mins)	**1Ah Pouch cell**
FCDAC//CNS[3]	0-4.2	40 (based on the weight of the electrode)	21000	coin cell
ANHCS//NHCS[4]	0.01-4	19.1	8203	coin cell
3DNFC//3DNFAC[5]	1.5-4.2	76.4	21000	coin cell
WS_2@NCN//NCHS[6]	0.6-4.2	103.4	235	coin cell
AC//$Ca_{0.5}Ti_2(PO_4)_3$@C[7]	1-4	34	5144	coin cell
U-Co_2P@rGO-14//AC[8]	1-4	87	4260	coin cell
AC//Sn[9]	1-4	120	2850	2-layer pouch cell
AC//SC[10]	0-4	120	599	coin cell
KTO//NGC[11]	0-3.5	58.2	7200	coin cell
WPCS//WPCS[12]	0-4.5	44	15400	coin cell
AC//$MoSe_2$/C-700[13]	1-4	106	588	coin cell
AC//NMCP@rGO[14]	0.01-4.2	63.6	19091	coin cell
AC//$NbSe_2$/NSeCNFs[15]	0.01-4	145	/	coin cell

Supplementary References

1. Augustyn V*, et al.* High-rate electrochemical energy storage through Li+ intercalation pseudocapacitance. *Nature materials* **12**, 518-522 (2013).
2. Kim H-S*, et al.* Oxygen vacancies enhance pseudocapacitive charge storage properties of MoO3− x. *Nature materials* **16**, 454-460 (2017).
3. Chen J*, et al.* Disordered, large interlayer spacing, and oxygen-rich carbon nanosheets for potassium ion hybrid capacitor. *Advanced Energy Materials* **9**, 1803894 (2019).
4. Qiu D*, et al.* Kinetics enhanced nitrogen-doped hierarchical porous hollow carbon spheres boosting advanced potassium-ion hybrid capacitors. *Advanced Functional Materials* **29**, 1903496 (2019).
5. Yang B*, et al.* 3D nitrogen-doped framework carbon for high-performance potassium ion hybrid capacitor. *Energy Storage Materials* **23**, 522-529 (2019).
6. Li Y*, et al.* Enhanced cathode and anode compatibility for boosting both energy and power densities of Na/K-ion hybrid capacitors. *Matter* **1**, 893-910 (2019).
7. Zhang Z*, et al.* Fast potassium storage in hierarchical Ca0. 5Ti2 (PO4) 3@ C microspheres enabling high-performance potassium-ion capacitors. *Advanced Functional Materials* **28**, 1802684 (2018).
8. Wang Y*, et al.* Ultrafine Co 2 P nanorods wrapped by graphene enable a long cycle life performance for a hybrid potassium-ion capacitor. *Nanoscale Horizons* **4**, 1394-1401 (2019).
9. Lang J, Li J, Ou X, Zhang F, Shin K, Tang Y. A flexible potassium-ion hybrid capacitor with superior rate performance and long cycling life. *ACS applied materials & interfaces* **12**, 2424-2431 (2019).
10. Fan L, Lin K, Wang J, Ma R, Lu B. A nonaqueous potassium-based battery–supercapacitor hybrid device. *Advanced materials* **30**, 1800804 (2018).
11. Dong S, Li Z, Xing Z, Wu X, Ji X, Zhang X. Novel potassium-ion hybrid capacitor based on an anode of K2Ti6O13 microscaffolds. *ACS applied materials & interfaces* **10**, 15542-15547 (2018).
12. Cui Y*, et al.* Bioinspired mineralization under freezing conditions: an approach to fabricate porous carbons with complicated architecture and superior K+ storage performance. *Acs Nano* **13**, 11582-11592 (2019).
13. Shen Q, Jiang P, He H, Chen C, Liu Y, Zhang M. Encapsulation of MoSe 2 in carbon fibers as anodes for potassium ion batteries and nonaqueous battery–supercapacitor hybrid devices. *Nanoscale* **11**, 13511-13520 (2019).
14. Ruan J*, et al.* Rational construction of nitrogen-doped hierarchical dual-carbon for advanced potassium-ion hybrid capacitors. *Advanced Energy Materials* **10**, 1904045 (2020).
15. Chen M*, et al.* An Ultrastable Nonaqueous Potassium-Ion Hybrid Capacitor. *Advanced Functional Materials* **30**, 2004247 (2020).
